# Advancing comparative placentation through spatial transcriptomics and organoid models

**DOI:** 10.1371/journal.pbio.3003346

**Published:** 2025-08-29

**Authors:** Francesca Soncin

**Affiliations:** 1 Department of Pathology, University of California San Diego School of Medicine, La Jolla, California, United States of America; 2 Center for Perinatal Discovery, University of California San Diego, La Jolla, California, United States of America; 3 Sanford Consortium for Regenerative Medicine, La Jolla, California, United States of America

## Abstract

More work is needed to characterize the evolutionary diversity of placental development in eutherian mammals. This Primer discusses a recent PLOS Biology study that provides a rich spatial transcriptomic characterization of the porcine placenta and develops a new swine trophoblast organoid model

## Teaser

The placenta is one of the most diverse organs among eutherian mammals, and comparative placentation studies can reveal fundamental processes that support a successful pregnancy. A new study in *PLOS Biology* uses swine placental organoids and *in vivo* spatial transcriptomics to uncover key insights into placental cell diversity and evolution.

The placenta is an evolutionary innovation of eutherian mammals, developed to sustain fetal growth within the maternal host [1]. It simultaneously performs endocrine signaling, modulates the immune system, regulates gas/nutrient/waste exchange, and serves as physical separation between the developing offspring and the maternal host. Despite its shared functions across species, the placenta is one of the most diverse organs, and the wide variation in placental architecture among mammals likely reflects adaptations to these demands. In this issue of *PLOS Biology*, McCutcheon and colleagues present a comprehensive characterization of cell populations at the swine maternal-fetal interface using a combination of spatial transcriptomics and trophoblast organoids derived from term placentae [2].

In humans and rodents, the hemochorial placenta is characterized by the erosion of maternal layers, allowing direct contact between maternal blood and the fetal-derived trophoblasts [1]. In contrast, species such as swine and horses exhibit epitheliochorial placentation, where all maternal and fetal cellular layers are retained, maintaining strict separation between the maternal and fetal compartments [1]. These structural differences between placentae across species raise fundamental questions: How do functionally equivalent outcomes arise from anatomically divergent designs? Can knowledge of the mechanisms of placentation from other species inform the development of diagnostics and treatment tools for human-specific placenta-associated diseases such as preeclampsia and gestational diabetes? In this context, comparative placentation studies are uniquely positioned to address such questions, offering insight not just into universal mechanisms of placental development, function, and dysfunction, but also into species-specific adaptations. Moreover, placental function influences both offspring and maternal health; therefore, a deeper understanding of its biology across species can offer invaluable insights for biomedical advances.

Placentation studies have been extensively conducted in humans and rodents due to their similar hemochorial placentation and the fast reproductive cycles of these animal models [3]. Novel platforms for single-cell, single-nuclei, and spatial transcriptomic analysis have provided new insights on trophoblast cell population diversity and tissue architecture [4]. Much less is known about trophoblast sub-populations in placentae of other non-traditional animal models, including swine. Moreover, novel models to investigate early placental development have recently been developed to overcome barriers such as general limitations in material abundance and ethical concerns in humans. In particular, organoids have proven to be scalable and tractable tools for studying complex biological systems [5,6]. In these models, progenitor cells spontaneously differentiate and self-organize into biologically relevant cell populations that recapitulate aspects of the original tissue, offering a more physiologically relevant model than traditional 2D cultures. Human trophoblast organoids derived from both first trimester and term placenta, as well as from trophoblast stem cell lines, are now widely used to investigate genetic and epigenetic mechanisms underlying normal placental development [7–9].

Following protocols previously established in the Coyne lab for the generation of human organoids [9], McCutcheon and colleagues derived swine trophoblast organoids (sTOs) from term porcine placentae. However, current knowledge on porcine trophoblast subtypes was insufficient to uniquely assign a specific cell identity to sTO cells identified by single-cell transcriptomic analysis. To address this, the researchers performed spatial transcriptomic analysis of the porcine maternal–fetal interface during mid-gestation. Using a combination of known markers, histology, and cluster-specific gene enrichment, they identified both maternal and fetal cell clusters, including interface and areolar trophoblasts. Subsequently, they used this spatial analysis of *in vivo* cell populations to guide the cell identity assignment of sTO cell clusters. They demonstrated that both interface and areolar trophoblasts were present in sTOs and that these cells maintained key functional pathways found in their *in vivo* counterparts.

Several aspects distinguish this study. First, the use of spatial transcriptomics provided anatomical resolution of cell populations and gene expression in swine placental tissue, a critical asset when studying organs with distinct functional regions. As in previous studies, spatial transcriptomics preserved architectural information that is otherwise lost in single-cell approaches [4]. Second, the derivation of organoids from term placentae overcomes the logistical and ethical constraints often associated with early gestation tissue collection, offering a scalable and reproducible platform for *in vitro* experimentation. Importantly, these 3D organoids reflected a diversity of trophoblast states not captured by traditional 2D cultures, expanding the toolbox available to placental biologists. However, the placenta is a dynamic organ throughout gestation, with trophoblasts undergoing progressive maturation over time [3]. Therefore, the extent to which trophoblast organoids derived from term placentas faithfully recapitulate early developmental mechanisms remains to be fully elucidated and warrants further investigation. Another limitation of the study is the largely descriptive and correlative nature of the findings. Similar to human and mouse studies based on large omics datasets, this work lacks mechanistic insight into the processes controlling swine placental development. Nonetheless, the derivation and validation of sTOs against primary tissue benchmarks provide a solid foundation for future mechanistic studies using this tractable model.

**Fig 1:**
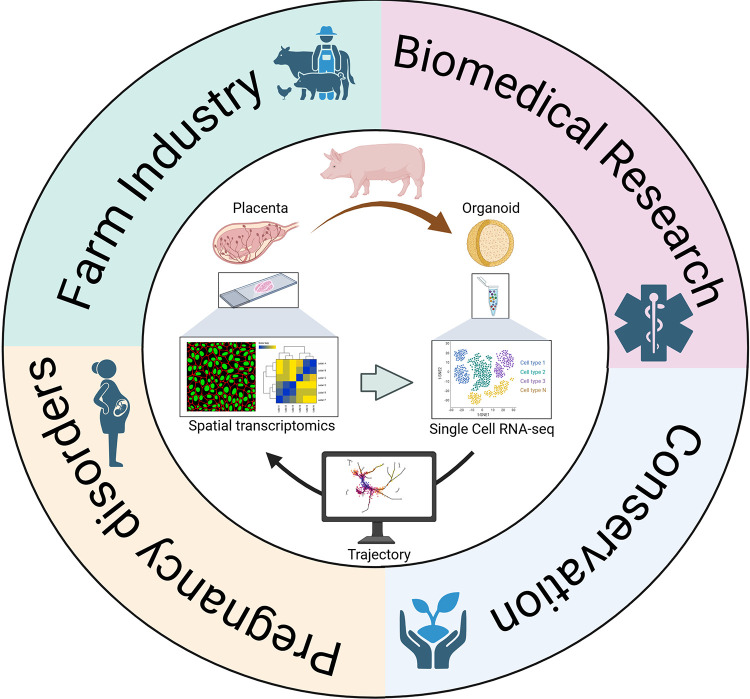
Implications of integrative omics studies of placental biology in non-traditional animal models on different fields. Created in BioRender. Soncin, F. (2025) https://BioRender.com/a7vtkg8.

The implications of this work are multi-faceted (Fig 1). In the specific context of swine placental biology, improving reproductive efficiency in livestock has substantial economic value. A better understanding of placental biology in pigs could optimize assisted reproductive technologies (ARTs) and enhance pregnancy outcomes, significantly reducing production costs. Moreover, ARTs are associated with a higher risk of placental dysfunction [10,11]. As ARTs gain traction in wildlife conservation efforts aimed at restoring viable populations of endangered species, these new tools and insights into placental development may be leveraged to improve reproductive outcomes, including ongoing global efforts to re-establish the extinct Northern white rhinoceros [12,13]. From a human health perspective, this comparative work is also compelling. First, pigs are frequently used in biomedical research. Thus, the economic and reproductive considerations described above for farming also apply in biomedical contexts. Furthermore, many human pregnancy complications, such as preeclampsia or placenta accreta, stem from defects in placental invasion and differentiation. While typically associated with hemochorial placentation, exploring alternative mechanisms in epitheliochorial species may reveal alternative compensatory pathways that might enhance gas and nutrient exchange in the context of mis-regulated invasion. This comparative lens could ultimately lead to novel therapeutic targets or biomarkers for diagnostics and management of human pregnancy disorders.

Finally, this study exemplifies a powerful strategy—combining omics approaches *in vivo* with physiologically relevant *in vitro* models such as organoids—to investigate both conserved and species-specific mechanisms of placental development and function across the animal kingdom. This integrated approach has the potential to inform both fundamental biology and applied science. As models like sTOs are established in additional species, they will undoubtedly illuminate the diversity and ingenuity of placental solutions shaped by millions of years of mammalian evolution.

## Abbreviations

ARTs: assisted reproductive technologies
sTOs: swine trophoblast organoids
